# Can the Framing of Climate Mitigation Actions into Government Policies Lead to Delivering Them? – Insights from Nepal’s Experience

**DOI:** 10.1007/s00267-022-01643-6

**Published:** 2022-04-23

**Authors:** Bishal Baniya, Prem Prakash Aryal

**Affiliations:** 1 Institute for Sustainable Futures, University of Technology Sydney, Sydney, NSW Australia; 2 Pokhara University, Nepal Engineering College, Bhaktapur, Nepal

**Keywords:** Climate mitigation, Energy and material consumption, Energy transition, Greenhouse gas emissions, Low-income country, Remittance

## Abstract

Many low-income countries (LICs), including Nepal, endeavour to deliver climate mitigation by reducing greenhouse gas (GHG) emissions and achieving more sustainable resource consumption. However, their prospects of delivering on such goals alongside the rapid structural changes in the economy prevalent in the LICs are not clear. This research aims to better understand the underlying complexity in the linkage between the framing of climate mitigation actions into government policies and the prospects for their delivery. We use critical discourse analysis, post-structural discourse analysis, and thematic analysis of textual data corpus generated from government policies (*n* = 12) and semi-structured interviews (*n* = 12) with policy actors, such as government policymakers and private sector and non-government organisations’ representatives. We also develop energy and material consumption and GHG emissions models to predict their values up to 2050 via the R tools and machine learning algorithms that validate the accuracy of models. Our findings suggest that the social context of policymaking creates a knowledge structure on climate mitigation which is reflected in government policies. The policy actors and their institutions exchange their ideas and interests in a deliberative and collaborative environment to prioritise policies for the energy, forest, and transport sectors to deliver climate mitigation actions in Nepal. However, the energy sector, together with the agriculture sector, has insufficient climate mitigation actions. Reflecting on the high proportion of biomass in the energy mix and the rapid rise in fossil fuel and energy consumption per capita—both of which are driven by the remittance inflows—this research suggests measures to reduce these in an absolute sense.

## Introduction

The international climate agreements and the environment-related Sustainable Development Goals (SDGs)—such as goals 7, 12, and 13—encourage policymakers from low-income countries (LICs) to frame climate mitigation actions into their government policies (Baniya et al. 2021a). Climate mitigation actions refer to both policy and non-policy actions^1^ that aim to reduce greenhouse gas (GHG) emissions, enhance the sustainable use of natural resources, improve resource efficiency^2^, and enable the renewable energy transition. The 2010 Cancun Climate Agreement had initially emphasised consideration of climate mitigation actions in developing countries via leveraging long-term financial options (e.g., Green Climate Fund), technology transfer between countries, and capacity building (UNFCCC 2010). Consequently, developing countries, including LICs such as Nepal, agreed to identify ways to address climate mitigation issues. However, we lack understanding about how LICs with low mitigative capacity (Ayers and Huq 2009), high vulnerability to the impacts of climate change (Dewan 2015), and low technical, financial, and institutional capacity (Busby and Shidore 2017; Tosun and Leininger 2017) address climate mitigation issues via government policies across policy formulation and implementation phases.

Our understanding of climate mitigation actions in LICs is limited because of the following two reasons. First, international climate conventions—up until the one in Copenhagen in 2009—focused on climate mitigation actions mainly in developed countries (UNFCCC 2010), which led LICs to consider climate mitigation as an agenda for developed countries and not for them (Ayers and Huq 2009). Therefore, there is insufficient empirical evidence on framing of climate mitigation actions in LICs’ government policies despite their recent interest in climate mitigation (Baniya et al., 2021b). Second, a deep-rooted structural impediment in LICs impairs their ability to deliver climate mitigation actions (UNCDP 2021). Structural impediment pertains to inadequate diversification of economic sectors, overdependence on least-productive economic activities, underutilisation of productive resources such as natural resources, human and financial capital, and widespread poverty (United Nations Economic and Social Commission for the Asia and the Pacific UNESCAP (2021); UNCTAD 2020). Dercon (2012) and Bariber (2016) had raised concern that these structural features of an economy act as barriers to implementing climate mitigation policies in LICs. To our knowledge, scholarly research focusing on climate mitigation in LICs has not explored climate mitigation actions’ delivery prospects alongside the structural impediments implicit in LICs. Thus, the insufficient consideration of the nuances of climate mitigation actions in LICs motivates this research to investigate the underlying complexity in the linkage between the framing of climate mitigation actions into government policies and the prospect for delivery.

Nepal has framed climate mitigation actions across government policies, formulated Climate Change Policy (2011 and 2019), and submitted Nationally Determined Contributions (NDCs) to the United Nations Framework Convention for Climate Change (UNFCCC) in 2016 and 2020. Therefore, Nepal is a good case country for this research, as the findings can be useful to other LICs. Nepal is amongst the most vulnerable countries because of its exposure to climate change risks such as floods, droughts, extreme heat, economy and human health, and other water-related impacts (Vinke et al. 2017). The high vulnerability to the potential impacts of climate change is unjust in a natural sense because Nepal’s contribution to the global GHG emissions was 0.04% of global GHG emissions in 2019 (Ritchie and Roser 2020). A tiny fraction of total global GHG emissions means that Nepal can continue its existing GHG emission trend under the contraction and convergence approach that allows countries with low per capita GHG emissions to seek equitable GHG emissions entitlements (Meyer 1999; Kuntsi-Reunanen and Luukkanen 2006). Nonetheless, despite its low GHG emissions per capita and low mitigative capacity (Ayers and Huq 2009; Baniya and Giurco 2021; Ritchie and Roser 2020), and the special circumstances as a LIC (Paris Agreement, Article 4.6), Nepal has demonstrated interest in reducing GHG emissions at economy-wide scale.

Laudari et al. (2021) examined Nepal’s first NDC formulation and implementation outcome to find that the country could not achieve most of its stipulated targets by 2020. However, the NDC document communicates signatory countries’ post-2020 climate mitigation actions, meaning Nepal’s NDCs are yet to be fully delivered. Therefore, it may not be reasonable to state that Nepal’s first NDC did not deliver the stipulated targets—rather, we need to know the possibility for delivering these climate mitigation actions by 2030 and beyond. In an intuitive sense, there is uncertainty about the future delivery of climate mitigation actions in Nepal, given the structural impediment and the technical, financial, and institutional constraints (UNCDP 2021). The discursive nature of the framing process further complicates the possibility of delivering climate mitigation actions (Baniya et al. 2021a). With these in the backdrop, this research investigates how framing is linked to the delivery, the discursive process involved in the policy formulation phase, and the evidence to suggest whether framing can contribute to the delivery of climate mitigation actions in Nepal. We build on the first-hand experience of government policymakers and non-government stakeholders, climate mitigation-related policy discourse, empirical findings from the government policies, and the quantitative evidence on resource use and GHG emissions in the context of the structural impediment in Nepal. The rest of the paper is structured as follows. Section “Theoretical foundations” is the theoretical foundation. Section “Methodology” explains the methodology. Section “Results and discussion” presents the results and discussions on key findings. Finally, Section “Conclusion” presents the conclusion.

## Theoretical foundations

Environmental policymaking—including the framing of climate mitigation actions into government policies in the global south (group of LICs)—depends on the global environmental discourse and the official development assistance (ODA) from international agencies (Biermann and Siebenhüner 2009; Karkee and Comfort 2016; Vij et al. 2018). These external factors have added extra responsibilities for LICs to focus on climate mitigation in addition to a traditional focus on climate adaptation. The international climate agreement and the associated international fund strongly influenced the NDC formulation process in Nepal (Laudari et al. 2021). The influence of global environmental discourse, its governance and the associated intergovernmental bureaucracies can be cognitive, normative, or executive (Biermann and Siebenhüner 2009). ‘Cognitive influences’ foster the informational basis of policies, such as the synthesis and dissemination of scientific knowledge about environmental problems. ‘Normative influence’ refers to the advancement of national and sub-national norm-setting for framing the agenda of global environmental discourse (e.g., climate mitigation actions) into government policies. ‘Executive influence’ relates to influencing policymaking during policy formulation and policy implementation by providing funding (Biermann and Siebenhüner 2009). This research presumes that the international climate agreements and the environment-related SDGs that are a part of global environmental discourse encourage a national policy paradigm that emphasises climate mitigation actions. A policy paradigm is a system of ideas that specifies policy goals and the instruments used to address specific problems (Hall 1993). Ideas refer to the contents, evidence, and values of policy actors, which originate from their interests during institutional interactions. Ideas, interests, and institutions are collectively referred to as ‘3Is’, which are key to explaining the policy change (Walt 1994; Shearer et al. 2016).

The interaction between policy actors’ ideas and their institutions, and the analytical distinction between these, are crucial to understanding policy change (Béland 2016). The conceptualisation of ideas as static and monolithic has led to a failure to sufficiently internalise the role of policy actors’ institutions in the policy change (Carstensen 2015). Kern et al. (2014) use the notion of an ‘interpretative framework of ideas’ to connect the ideational element of policymaking with the institutionalist perspective on policy change because the policy actors’ institutions embed the interpretative framework of ideas. The ways formal institutions, such as governmental organisations, work on their mandates—and the ways institutions interact and operate—present an interpretative framework of ideas as an important aspect of policy paradigms that influences policy goals and instruments (Kuzemko 2013, p. 48). The interaction between policy actors’ ideas and institutions is profound enough to discuss these collectively, reflecting on the ‘discursive institutionalism’ that takes ideas and institutions seriously, sets ideas and discourse into institutional contexts, and identifies these as having dynamic characteristics (Schmidt 2008). After the advent of discursive institutionalism, the link between ideas and institutions appeared to be more prominent in policy studies, and the positive aspects of the linkage, in particular, are discussed widely. However, Carstensen and Schmidt (2016) think that ideational power and the capacity of actors to influence others’ cognitive beliefs may result in institutions imposing their agendas on others. This may be true in a multi-level governance setting where central government institutions have more power over local government institutions. This research focuses on the interactions between policy actors across sectors and multi-level governance and their institutions to understand the ways in which the policy actors share ideas about the global environmental discourse via their institutions.

While climate mitigation actions in government policies aim to achieve reductions in GHG emissions and enhance sustainable use of resources across economic sectors, there remains a question—are policy actions from LICs that are insignificant contributors to global GHG emissions required? Turner (2014, p. 5) found that policy issues relating to climate mitigation have motivated governments in developing Asian countries to promote energy security, access to clean energy, protect forest resources, pursue technological advantage, and address local environmental problems. Another non-climate benefit is that LICs can generate revenues from selling emissions permits to developed countries under mechanisms related to a contraction and convergence regime, which promotes fair distribution of GHG emissions per capita (Hübler 2011). This research uses notions of non-climate and local benefits to study the framing of climate mitigation actions in a country with a low GHG emissions mitigative capacity.

## Methodology

This research uses both qualitative and quantitative research methods. Critical Discourse Analysis (CDA), Post-structuralist Discourse Analysis (PDA), and Thematic Analysis (TA) are the qualitative research methods. We also use the quantitative research method to create models that predict the future energy and material consumptions and GHG emissions values. The use of the mixed method can be challenging because of the multiple data collection phases and different data procedures employed, but it can help test the consistency of findings and build on the results of one method with another (Rogers et al. 2003; Wheeldon 2010). While the use of the constructivist approach is prevalent in policy analysis, particularly during agenda-setting, the discourse analysis (such as CDA and PDA) are rarely connected to empiricist and positivist approaches (e.g. quantitative research methods) because of the ontological and epistemological tensions (Leipold et al. 2019). Our methodological design builds on the idea that constructivist, empiricist, and positivist epistemological paradigms can go together. Thus, we seek a connection between the discursive nature of non-material ideas and interests of policy actors and the material issues pertaining to the sustainable use of natural resources and GHG emissions.

### Qualitative research methods

The constructivist epistemological paradigm adopted by this research focuses on three crucial aspects of framing climate mitigation actions into government policies. First, factors that create a context for framing climate mitigation actions in a country that has historically emphasised climate adaptation actions and is also one of the least contributors to the global GHG emissions. Thus, it is imperative to study how a base for a radical shift in climate change-oriented policies is created. We use CDA and investigate elements such as discursive differences, preferential values of policy actors, and differing policy actors’ ideologies and ideas in general (Supplementary Table 1). Second, whilst framing climate mitigation actions, the institutional approach may be different for different institutions as some may prioritise climate mitigation actions, and others may see them as secondary issues. Thus, the institutional perspective to framing climate mitigation actions is also important. It explains the interaction between different responsible government and non-government institutions, which we study using PDA. The PDA focus on elements such as institutional interest-based discourse, ideational power of one institution over another, and the directionality of institution in terms of responsibility in multi-level governance (Supplementary Table 1). Finally, while framing climate mitigation action is a part of environmental policymaking, the possibility of delivering climate mitigation actions on the ground depends on various enablers. What can be the various enablers, particularly for LICs that lack resources at various fronts and levels? To answer this question, we use TA and study three elements, such as capacities across different fronts (technical, financial, and institutional), determinants for delivery of climate mitigation actions, and stakeholders. The Supplementary Table 1 explains the description and purpose of each qualitative research method.

Government policy documents (*n* = 12) focusing on climate mitigation actions are the data source for CDA and PDA. Following are the government policy documents chosen based on their consideration of climate mitigation actions as policy objectives, based on policies of different economic sectors, and based on implementation stage: Climate Change Policy 2011 (Ministry of Forest and Environment MoFE 2021a); Industrial Policy 2011 (Ministry of Industry, Commerce, and Supplies MoICS 2011); National Energy Strategy 2013 (WECS 2013); National Sustainable Transport Strategy 2015 (Ministry of Physical Infrastructure and Transport MoPIT 2015); Nepal Reducing Emissions from Deforestation and Forest Degradation (REDD+) Strategy 2015 (Ministry of Forest and Soil Conservation MoFSC 2015); Agriculture Development Strategy 2015 (Ministry of Agriculture Development MoAD 2015); First Nationally Determined Contribution 2016 (Ministry of Population and Environment MoPE 2016a); Renewable Energy Subsidy Policy 2016 (MoPE 2016b); Forest Sector Strategy 2016 (Ministry of Forest and Soil Conservation MoFSC 2016); Nepal Agroforestry Policy 2019 (Ministry of Forest and Environment MoFE 2019); National Climate Change Policy 2019 (Ministry of Forest and Environment MoFE 2021); and Second Nationally Determined Contribution (MoFE 2020). We create a textual data corpus from the chosen policies, which we analyse in the interactive and social context of policymaking in Nepal because CDA sees language (written texts) as social practice (Fairclough and Wodak 1997; Wodak 2001) and context-sensitive (Huckin 1997). The emphasis on social practice that shapes discursive events, such as policymaking, is justified in the sense that policy per se is socially constructed (Bacchi 1999). While generating a textual data corpus, we emphasise certain concepts for further foregrounding and de-emphasise others based on the research problem that needs to be defined in CDA (Keller 2013, p. 27–28).

We intended to unlock information from the manifestations of discourses on framing climate mitigation actions into government policies and to discover how framing is achieved across policies of different economic sectors. We were also interested to know how government policies changed by using the notion of a climate change-related policy paradigm that emphasises policy problems, policy goals, and policy instruments (Vij et al. 2018; Baniya et al. 2021b). The discursive differences are negotiated, and differences in power are encoded in texts, meaning the text corpus can show traces of differing ideologies and discourses (Wodok and Meyer 2001). In this way, we analyse the textual data corpus, which is the product of discursive practice in policymaking (Huckin 1997) to interpret findings in a broader social context beyond public policy and explain the social processes and knowledge structures that generate texts (i.e., government policies).

Semi-structured interviews (*n* = 12) with federal and local level government policymakers, private sector organisations (industry associations), and non-government international development organisations provide textual data for PDA and TA. A predetermined order of questions used in structured interviews rarely contributes to the gathering of different ideas and opinions from a diverse group of research participants. On the other hand, semi-structured interviewing is flexible because it can include situation-specific open-ended questions that can generate additional insights into the topic at hand. A purposive sampling technique is used for the semi-structured interviews (SSIs). We base the use of purposive sampling on the following criteria: 1) respondents have solid hands-on experience in environmental policymaking and have a few years of experience in the field of climate change, 2) respondents work for the government, private sector, non-government, and international development organisations, 3) almost half of the respondents have an educational/professional background in social science and/or economics, and 4) understand national and local level climate change policy landscape as demonstrated by their current role.

Research that is not concerned with statistical generalisability often uses non-probabilistic sampling, such as purposive sampling, to select participants according to predetermined criteria relevant to a specific research objective (Vogel et al. 2002). The main inclusion criteria for the research participants were that they had experience in policymaking, and that they were affiliates of an active government, private sector, or non-government organisation in Nepal. Regarding the number of research participants for interviews, Guest et al. (2006) argue that data saturation often occurs after around 12 interviews. For phenomenological studies, Guest et al. (2006) found the following: “Morse (1994, p. 225) recommends at least six participants; Creswell (1998) recommends between five and twenty-five interviews; whereas Kuzel (1992, p. 41) emphasises heterogeneous sample and research objectives to recommend six to eight interviews. For in-depth interviews in a naturalistic setting, having a small sample size (less than 20) can be a practical way to engage with the research participants while exchanging information openly (Crouch and Mckenzie 2006). We chose 12 participants for the semi-structured interviews, as this number is assumed to be reasonable for generating meaningful insights from the in-depth discussion.

Dunn (2001) used various written texts and semi-structured interviews as data sources for discourse analysis (Waitt 2005). This research’s use of PDA is based on similar data sources, which are government policies and semi-structured interviews. PDA is primarily for understanding the interaction between the policy actors and their ideas and institutions, the discourses based on policy actors’ interests, the directionality of institutional interactions in multi-level governance decision-making, and the presence of any argumentative turn. Directionality of institutional interaction refers to how one or more institutions exercise power to influence other institution(s) across cross-sectoral and multi-level governance. While discursive institutionalism can help understand the relationship between ideas, discourse, and institutions, PDA is particularly helpful in understanding the power dimension of the discourse (Torfing 1999, p.153; Panizza and Miorelli 2013) and the intersubjective aspect of ideas (Oscar and Larsson 2015).

As part of using TA, this research uses the description and interpretation of research participants to examine the following three specific issues; first, the capacity to frame climate mitigation actions into government policies divided into institutional, financial, and technical capacity; second, consideration of key determinants for delivering the climate mitigation actions via policy implementation; third, the key stakeholders in both framing and delivering climate mitigation actions. While the use of TA has been questioned because it focuses on the explicit description (Smith et al. 2011; Vaismoradi et al. 2013), we focus on both the explicit and implicit meaning of the textual data generated from the semi-structured interviews. A content-sensitive analytical approach to systematically assessing the manifest and latent contents of the textual data and contextual information enhances validity and rigour (Selvi 2020, p. 81). We use a constructionist approach to create themes based on the aforementioned three specific issues, and these are determined in advance of full analysis (Guest et al. 2012). Themes are further divided into codes in NVivo software, where relevant textual data from semi-structured interviews are added. For an epistemological study that uses previous literature, Graneheim et al. (2017) recommend analysing the manifest content first and then analysing the latent content to harness the implicit meaning, which is also a basis for interpretation of manifest contents.

### Quantitative research method

We use quantitative predictive modelling to create Total Energy Consumption (TEC), Domestic Material Consumption (DMC)^3^, and GHG emissions models for Nepal. The three models are used to predict their values up to 2050. For TEC and DMC models, we take gross domestic per capita (GDP per capita), remittance (Rem), and official development assistance (ODA) as independent variables. The relationship between energy consumption, GHG emissions, and GDP per capita has been thoroughly explored by econometric studies focusing on Nepal and other countries (Lee and Chang 2007; Pokharel 2007; Begum et al. 2015; Vidyarthi 2015; Bastola and Sapkota 2015). The relationship between energy consumption and remittance is also explored by previous studies (Yang et al. 2020; Akçay and Demirtaş 2015; Rahman et al. 2021; Das and McFarlane 2020; Sharma et al. 2019), focusing on high remittance-receiving South Asian countries. However, the relationship between ODA and energy consumption is relatively less explored as this body of literature has only recently started to receive attention (Sharma et al. 2019; Carfora et al. 2021). The causal relationship between remittances and energy resources (e.g., biomass and fossil fuels), and between ODA and the same energy resources are explored thoroughly by Baniya and Aryal (2022) with Nepal as a case country. They found that both remittances and ODA can drive consumption of energy resources such as biomass and fossil fuels and has both short-run and long-run causal relationship. Biomass and fossil together contribute to more than 85% of the total energy consumption in Nepal (Baniya and Aryal 2022; WU 2019), and therefore, it is reasonable to view both remittances and ODA as drivers to the total energy consumption in Nepal.

The relationship between material consumption and GDP per capita, remittance, and ODA is also less studied (Baynes and Musango 2018; Popescu et al. 2019; Sarkodie 2020). Most of the research exploring the relationship between material consumption and economic output (e.g., GDP) uses the concept of resource decoupling, which is the decoupling of material use from GDP (Haberl et al. 2020). A Google Scholar and the Scopus database search showed a single occurrence of the DMC model for Nepal (Baniya et al. 2021c) that has taken GDP and population as independent variables. Thus, the addition of two more independent variables—i.e., remittance and ODA—will generate more insights into the role of external financial inflows in the DMC of Nepal. This will also shed light on material consumption under an economy’s structural and macroeconomic changes. Further, Baniya and Aryal (2022) found that remittances and ODA cause the consumption of materials such as biomass, fossil fuels, non-metallic minerals, and metal ores for Nepal. These individual material types had both short-run and long-run causal relationships with remittances and ODA. This study looks at the total domestic material consumption and builds on the findings of Baniya and Aryal (2022) to further investigate domestic material consumption in the context of climate mitigation-oriented policies. The domestic material consumption of Nepal is largely biomass-based (WU 2019), and therefore, any changes in the energy system, notably the rural energy transition (e.g. use of the solar home system, micro-hydro, and biogas) driven by remittances and ODA inflows (Das et al. 2021) will contribute to the nation’s climate mitigation goals.

For the GHG emissions model, we use agriculture, forestry, and fishing’s share of GDP as an independent variable for its significant contribution to the total GHG emissions in Nepal, which is about half of the nation’s GHG emissions currently, compared with more than two-thirds about a decade ago. The GHG emissions from agriculture in Nepal come primarily from enteric fermentation, manure management, managed soil, rice cultivation, and field burning, whereas the forest land removes GHG emissions through natural biomass growth (Ministry of Science Technology Environment MOSTE 2014; Ministry of Forest and Environment MoFE 2021). The reduced share of the agriculture sector (including forestry and land use) in the nation’s total GHG emissions has been compensated for mainly by the energy sector, owing to the increased use of fossil fuels and the decrease in the share of renewable energy in the energy mix of Nepal (World Bank 2021). Between 1985 and 2017, there was an almost eleven-fold increase in fossil fuel use in Nepal despite a marginally less than three-fold increase in the TEC during the same period (WU 2019; IEA 2021). Therefore, we take the total energy consumption per capita as an independent variable for the GHG emissions model. Pradhan et al. (2017) have modelled GHG emissions for Nepal using the AFOLU-B model that is individual sector-focused. We account for the decreasing agriculture, forestry, and fishing sector’s share in the GDP and the significant rise in energy consumption use per capita over the last two decades. These two independent variables, together with the GDP per capita, consider the impact of both macroeconomic and structural changes in the economy and on the nation’s total GHG emissions. While the percentage contribution and absolute GHG emissions from the energy sector are on the rise, absolute GHG emissions from the agriculture sector (including forestry and land use) are also on the rise and is projected for marginal increase despite the reduced share of agriculture, forestry, and fishing in Nepal’s GDP (World Bank 2021; Ministry of Forest and Environment MoFE 2021). This is mainly because Nepal’s GDP from agriculture, forestry, and fishing has increased four-fold in the last two decades (World Bank 2021). Thus, we take into the structural dynamics of Nepal’s economy, and the way changes in the economy’s structure influence the overall GHG emissions at a nation-wide scale. We base our logic to choose the share of agriculture, forestry, and fishing’s share of GDP as an independent variable on the above mentioned economy’s structural changes.

We source the variables’ time-series data from the development indicator database of the World Bank (World Bank 2021) and materialflows.net (WU 2019). For TEC and DMC models, we use the variables’ data from the period between 1993 and 2019. For the GHG emissions model, the period is between 1990 and 2019. While the TEC and GHG emissions models have been developed by previous studies focusing on Nepal, there is a lack of research about the DMC model for Nepal (Baniya et al. 2021c). We also focus on the methodological contribution by using regularised regression method—ridge regression—that employs a machine-learning approach to create models with the least error and use cross-validation algorithms to test the validity of the models. Section “Ridge regression and models” explains the use of ridge regression in detail.

#### Ridge regression and models

**Fig. 1:**
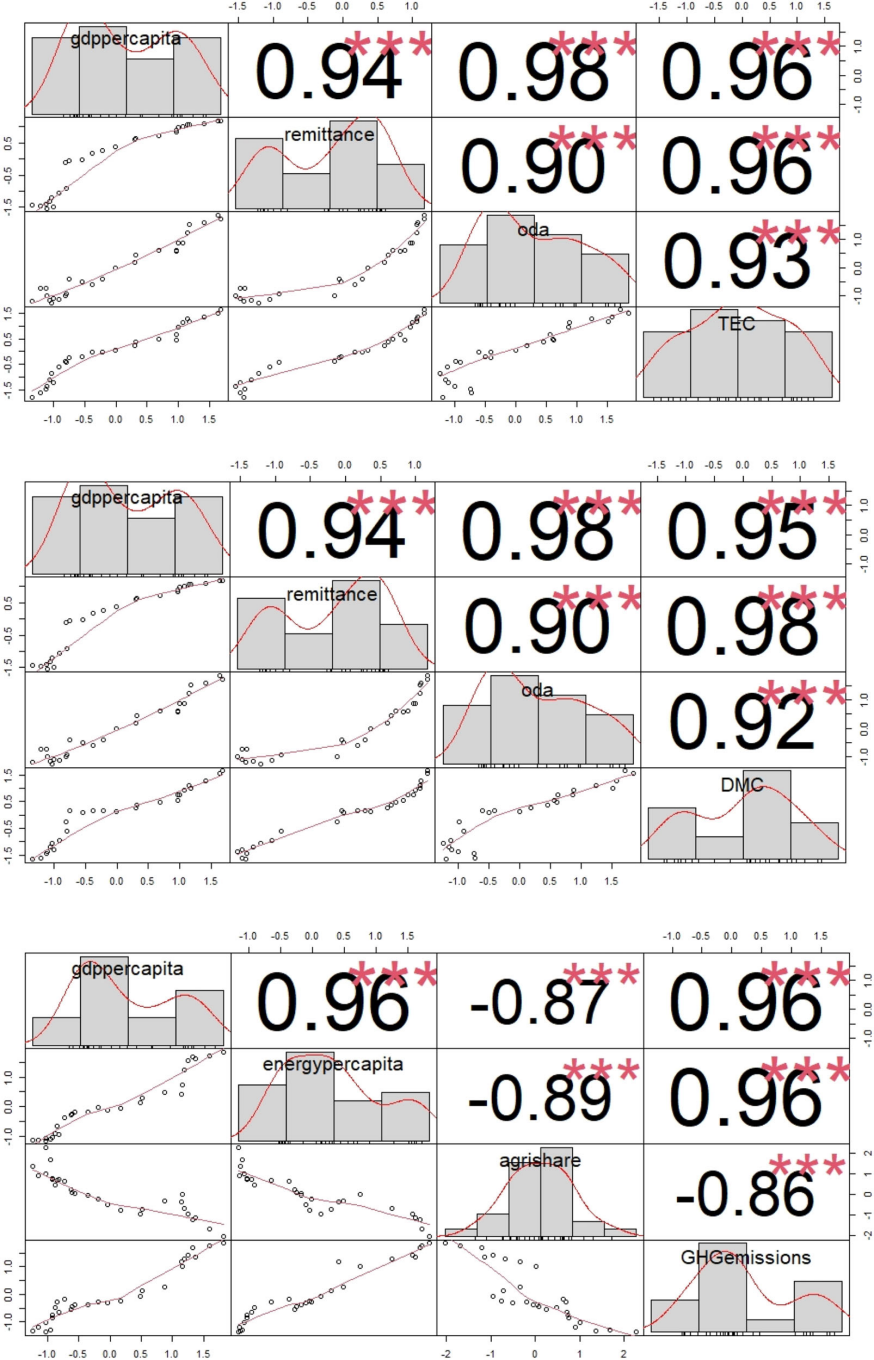
Variables distribution (diagonal), the bivariate scatter plots with fitted lines, and correlation values with significance levels (***p < 0.05)

We chose the ridge regression method for two main reasons. First, we want to minimise standard errors from multicollinearity between independent variables (Fig. 1 shows the variables’ distributions, bivariate scatter plots, and the correlation values with significance levels, which are less than 0.05 for all variables. The linear relationship and high correlation between independent variables indicate that there is a presence of multicollinearity.) Second, to seek a balance between low variance and low bias, which is often achieved by using regularised regression methods, such as ridge regression. It avoids a significant change in the coefficient estimates from a small change in a single data point of any independent variable. Ridge regression does so by incorporating a regularised parameter, which penalises large coefficients, thus reducing the sum of squared residuals and the complexity of the model. The regularised parameter is referred to as ‘shrinkage penalty’ and is denoted by λ in this paper.

We construct ridge regression models for TEC, DMC, and GHG emissions by using the following equations. We use a logarithm scale for each variable to reduce the impact of heteroscedasticity on the constructed models.

1 $$\begin{array}{ll}{{{\mathrm{ln}}}}\left( {{{{\mathrm{TEC}}}}} \right) = \beta _0 + \beta _1{{{\mathrm{ln}}}}\left( {{{{\mathrm{GDP}}}}/{{{\mathrm{capita}}}}} \right)\\ + \beta _2{{{\mathrm{ln}}}}\left( {{{{\mathrm{Rem}}}}} \right) + \beta _3{{{\mathrm{ln}}}}\left( {{{{\mathrm{ODA}}}}} \right) + \mu\end{array}$$

2 $$\begin{array}{ll}{{{\mathrm{ln}}}}\left( {{{{\mathrm{DMC}}}}} \right) = \beta _0^\prime + \beta _1{{{\mathrm{ln}}}}\left( {{{{\mathrm{GDP}}}}/{{{\mathrm{capita}}}}} \right)\\ + \beta _2^\prime {{{\mathrm{ln}}}}\left( {{{{\mathrm{Rem}}}}} \right) + \beta _2^\prime {{{\mathrm{ln}}}}\left( {{{{\mathrm{ODA}}}}} \right) + \mu^\prime\end{array}$$

3 $$\begin{array}{ll}{{{\mathrm{ln}}}}\left( {{{{\mathrm{DMC}}}}} \right) = \beta _0^\prime + \beta _1{{{\mathrm{ln}}}}\left( {{{{\mathrm{GDP}}}}/{{{\mathrm{capita}}}}} \right)\\ + \beta _2^\prime {{{\mathrm{ln}}}}\left( {{{{\mathrm{Rem}}}}} \right) + \beta _2^\prime {{{\mathrm{ln}}}}\left( {{{{\mathrm{ODA}}}}} \right) + \mu^\prime\end{array}$$

where ‘TEC’ is the total energy consumption; ‘GDP/capita’ is the GDP per capita; ‘Rem’ is the remittance; ‘ODA’ is the official development assistance; ‘TEC/capita’ is the total energy consumption per capita; and the ‘agrishare’ is the agriculture, forestry, and fishery sector’s share in the GDP. β values are the coefficients of the independent variables, and µ values are errors.

We use both standardised and unstandardised variables for statistical testing by using the R tool (R Core Team 2017) and its packages such as the glmnet, ggplot2, and corrgram (Friedman et al. 2010; Wickham 2016; Friendly 2002). We split the variables’ dataset into training matrices and testing matrices as 70% and 30% of the total dataset, respectively. The split of variables’ dataset into training and testing, together with the use of the K-fold cross-validation method, allows for the testing of the model by creating K = 10 models and running the code to find the optimal value of the shrinkage penalty (λ) that corresponds to the lowest test mean squared error. Whilst using the K-fold cross-validation method, we chose K = 10 for an optimal balance between variance and bias. The value of K between 5 and 10 yields estimates of test error that do not suffer from high bias and high variance (Gareth et al. 2013, p. 184).

#### Scenarios

Whilst this paper uses unstandardised coefficients to predict TEC, DMC, and GHG emissions values up to 2050 by using Eq. (1), Eq. (2) and Eq. (3), respectively, we do not intend to pinpoint the future values exactly. Instead, we consider the potential fluctuations in the independent variables’ data values in the future. Therefore, we aim to predict the future values of TEC, DMC, and GHG emissions in a range by creating three scenarios, which are: 1) Existing Trend Scenario (ETS); 2) Low-Value Scenario (LVS); and 3) High-Value Scenario (HVS). Table 1 shows the scenarios, descriptions, and the independent variables’ data values for each of the three dependent variables (TEC, DMC, and GHG emissions). In ETS, we assume that the existing trend of growth (GDP per capita), external financial inflows, energy consumption per capita, and agriculture sector share in the GDP will continue until 2050. We define the existing trend as the trend in the period between 2009 and 2019. In LVS, we assume that the existing trend will break, and the independent variables will take their lowest values between 2009 and 2019. Finally, in HVS, we assume that the independent variables will take their highest values between 2009 and 2019. LVS and HVS will allow us to predict TEC, DMC, and GHG emissions values in a range. It will also reduce the complexity of multiple scenarios based on possible future changes in each independent variable. Whilst choosing the independent variables’ data, we considered the exclusion of outliers and the impact of COVID-19 because the country is expected to have a positive economic growth rate in 2021 and beyond (Asian Development Bank ADB 2021) despite an economic slowdown in 2020.

**Table 1:** Scenarios and their description

Scenarios	Description	TPEC and DMC	GHG emissions
*Existing trend scenario (ETS)*	Existing trend continues and the independent variables (GDP per capita, remittances, ODA, and agriculture’s share in an economic output) take their average annual growth rate from the last decade	GDP per capita (4.75%), Remittance (12.4%), ODA (9.9%)	GDP per capita (4.75%), TPEC per capita (2.3%), Agriculture’s share in economic output (−2.6%)
*Low value scenario (LVS)*	The independent variables take their low growth rate values from the last decade	GDP per capita (2.9%), Remittance (4.8%), ODA (2.7%)	GDP per capita (2.9%), TPEC per capita (1.5%), Agriculture’s share in economic output (−7%)
*High value scenario (HVS)*	The independent variables take their high growth rate values from the last decade	GDP per capita (6.8%), Remittance (16.5%), ODA (19%)	GDP per capita (6.8%), TPEC per capita (4%), Agriculture’s share in economic output (−3%)

## Results and discussion

We present two different types of results and discuss them collectively. First is the quantitative modelling of TEC, DMC, and GHG emissions and their predicted values up to 2050 for three scenarios. The results from the modelling and scenarios highlight the delivery prospect of climate mitigation actions and its relationship with the economic factors (e.g., GDP per capita, remittance, and ODA) and the structural features of the economy, resource use, and GHG emissions (e.g., the agriculture, forestry, and fishery sector’s share in the GDP and the TEC per capita). The second is the qualitative results from CDA, PDA, and TA. These highlight the manifestations of climate mitigation actions across government policies, the way climate mitigation actions are deliberated for framing by policy actors, and the way policy actors’ institutions leverage their power in a discursive policy environment. Based on these findings, we discuss the important linkage between the framing and delivery of climate mitigation actions in the context of LIC. In this way, we generate insights into whether the framing of climate mitigation actions and the way policy actors envision their delivery is fragmented.

### Results from the qualitative empirical analysis and predictive modelling

**Fig. 2:**
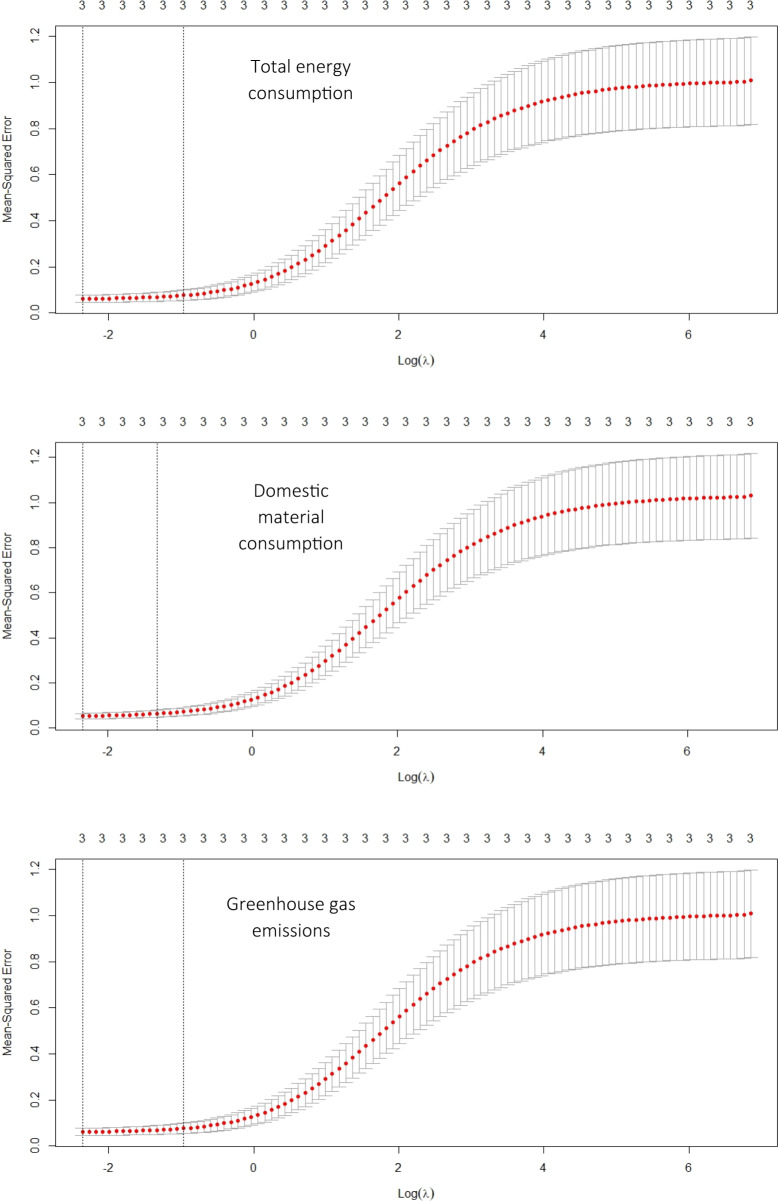
The shrinkage penalty (λ) value that minimises the mean squared error

**Fig. 3:**
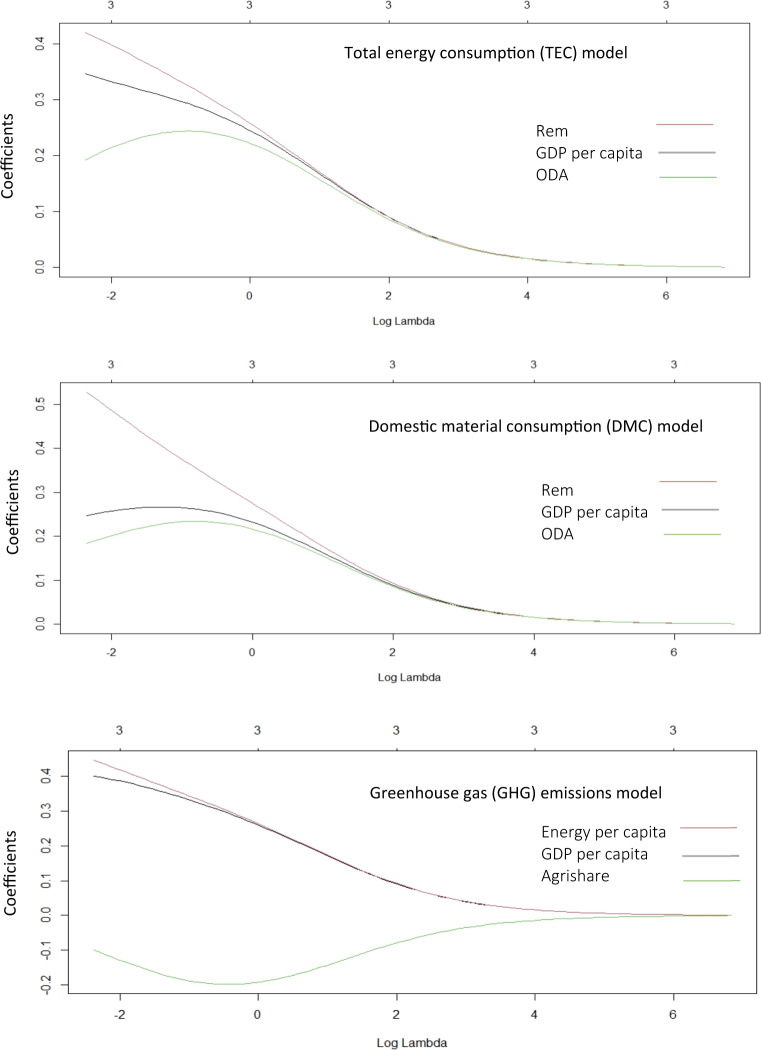
Ridge trace plot to visualise the changes in standardised coefficients’ estimates as the shrinkage penalty (λ) chose its optimal value by minimising the mean square error

Tables 2 and 3 show the unstandardised and standardised coefficients of the independent variables, the optimal shrinkage penalty, and the optimal cross-validated sum of squared residuals for TEC, DMC, and GHG emissions models. Figure 2 shows the shrinkage penalty (λ) value that minimises the mean squared error. Figure 3 shows the ridge trace plot to visualise the changes in standardised coefficients’ estimates as the shrinkage penalty (λ) chose its optimal value by minimising the mean square error. For TEC and DMC models, the remittance effect is the strongest, followed by the GDP per capita and the ODA. For every two units increase in remittance, TEC and DMC will see almost a unit increase, meaning that remittance strongly drives energy and material consumption in Nepal. In absolute terms, remittance has increased by almost eight-fold. In terms of remittance’s share of GDP, it has increased by almost ten-fold (World Bank 2021). Reflecting on this trend of rising remittance, a further increase in energy and material consumption can be expected in the future. Likewise, the GDP per capita and ODA have a positive effect on energy and material consumption. Almost a three-unit increase in GDP per capita is required for a unit increase in energy consumption. Almost a five-unit increase in ODA is required for a unit increase in energy consumption if other variables remain constant. For a unit of material consumption, almost a four-unit increase in GDP per capita and a six-unit increase in ODA is required. In the last two decades, Nepal has achieved an almost five-fold increase in GDP per capita and a four-fold increase in the ODA, and these appear to drive the energy and material consumption in Nepal.

**Table 2:** Energy and material consumption models

		GDP per capita (GDP/cap)	Remittance (Rem)	Official Development Assistance (ODA)	Constant	Optimal shrinkage penalty (λ)	R2 (optimal cross-validated sum of squared residuals)
Total Energy Consumption	Unstandardized coefficients	0.124	0.046	0.078	8.54	0.008	0.948
	Standardised coefficients	0.346	0.423	0.191	0	0.094	0.947
Domestic Material Consumption	Unstandardized coefficients	0.118	0.078	0.101	6.015	0.012	0.955
	Standardised coefficients	0.245	0.530	0.184	0	0.095	0.955

**Table 3:** Greenhouse gas (GHG) emissions models

		GDP per capita (GDP/cap)	Energy per capita (Energy/cap)	Share of Agriculture (agrishare)	Constant	Optimal shrinkage penalty (λ)	R2 (optimal cross-validated sum of squared residuals)
Greenhouse gas (GHG) emissions	Unstandardized coefficients	0.205	1.068	−0.200	−1.446	0.010	0.936
	Standardised coefficients	0.399	0.449	−0.098	0	0.094	0.940

**Fig. 4:**
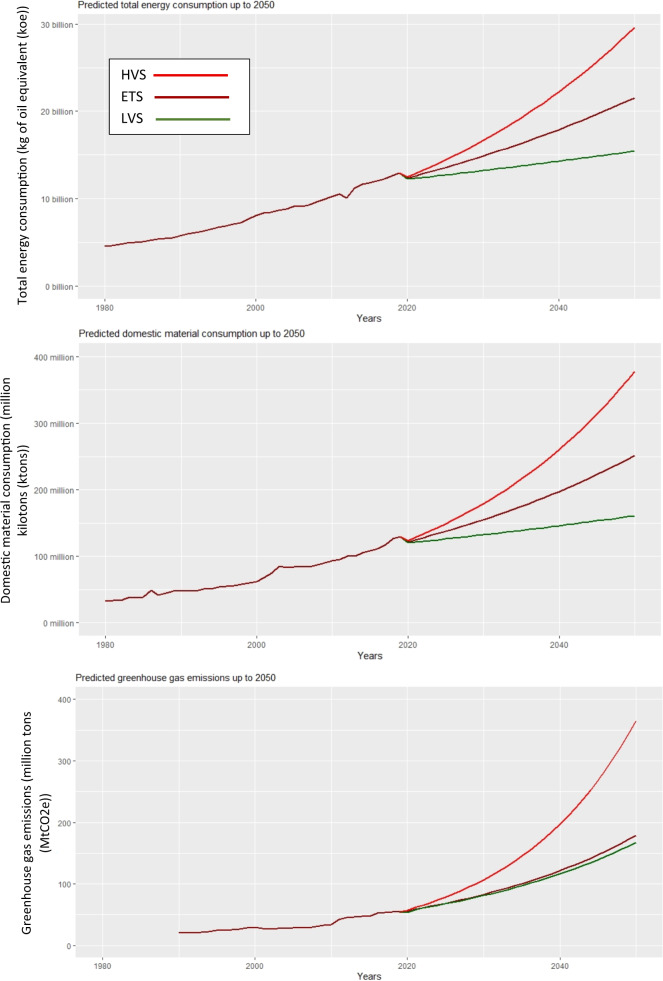
Predicted trend of total energy consumption, domestic material consumption and greenhouse gas emissions in Nepal up to 2050

For GHG emissions, the energy consumption per capita appears to be the strongest factor followed by the GDP per capita as both have a positive effect on the GHG emissions, meaning an increase in any of these two variables will cause an increase in GHG emissions. On the other hand, the share of the agriculture, forestry, and fishery sector in the GDP appears to have a negative effect on GHG emissions. However, a unit increase in either GDP per capita or energy consumption per capita will cause more change (increase) in GHG emissions than a unit decrease in the share of the agriculture, forestry, and fishery sector in the GDP. For this reason, GHG emissions are expected to rise more sharply than the TEC and DMC (Fig. 4).

Figure 4 shows the projected trend of TEC, DMC, and GHG emissions in absolute terms for three scenarios—the Low-Value scenario (LVS), the Existing Trend Scenario (ETS), and the High-Value Scenario (HVS). Table 4 shows the projected values of TEC, DMC, and GHG emissions for three scenarios in 2030, 2040, and 2050. While the absolute values of TEC, DMC, and GHG emissions will increase in 2030, 2040, and 2050 with respect to 2019 for all scenarios, GHG emissions in HVS shows a contrastingly sharp rise (almost seven-fold increase by 2050) because of the high GDP per capita and energy consumption per capita growth rates. In LVS and ETS, the GHG emissions will see a steady rise with a possibility of an almost three-fold increase in GHG emissions by 2050 with respect to 2019. While the low growth rates across all independent variables are likely as in the LVS scenario, their recent trend in the last two decades indicates that the GHG emissions value in 2050 may range between ETS and HVS values (i.e. 180–365 MtCO2e). Between 1990 and 2019, GHG emissions grew by only about one-fold, which indicates that the rapid rise in the energy consumption per capita and the associated GHG emissions from the energy sector will be far more significant than the historical contribution of the agriculture, forestry, and fishery sector in the nation’s GHG emissions. Therefore, even though the current share of GHG emissions from the agriculture, forestry, and fishery sector is about half of the nation’s GHG emissions, policies focusing on reducing GHG emissions from the energy sector appear to be important for the future. This finding indicates that LICs will follow the developed countries in terms of generating the majority of GHG emissions from the energy sector.

**Table 4:** Energy and material consumption and GHG emissions values for different scenarios up to 2050

	2019	2030			2040			2050		
		Low-Value Scenario (LVS)	Existing Trend Scenario (ETS)	High-Value Scenario (HVS)	Low-Value Scenario (LVS)	Existing Trend Scenario (ETS)	High-Value Scenario (HVS)	Low-Value Scenario (LVS)	Existing Trend Scenario (ETS)	High-Value Scenario (HVS)
Total energy consumption (billion koe)	12.9	13.2	14.8	16.6	14.3	17.9	22.2	15.5	21.5	29.6
Domestic material consumption (million ktons)	129.7	132.2	155	179.2	145.8	197.5	260.3	160	251.6	378.1
Greenhouse gas emissions (million tons)	54.3	80.9	82.9	106.8	116.3	121.8	197.4	167.2	178.9	365

**Fig. 5:**
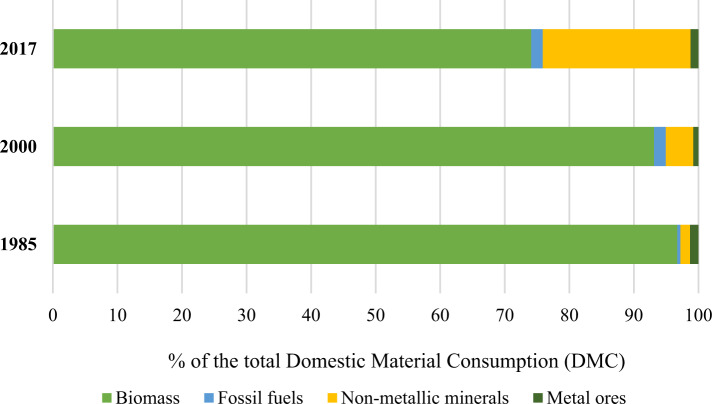
% of the total domestic material consumption (Data source WU 2019)

**Fig. 6:**
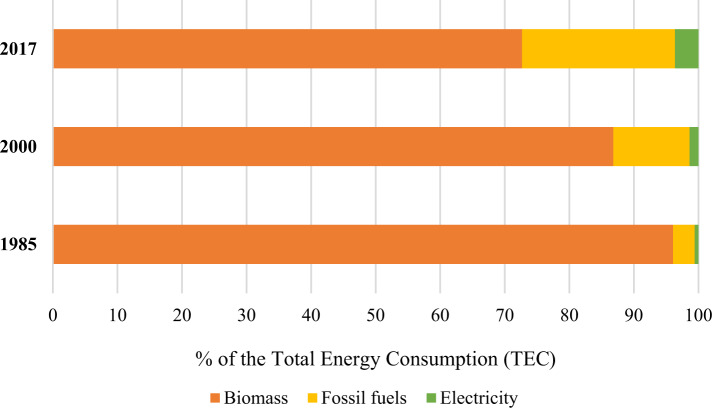
% of the total energy consumption (Data source IEA 2021)

The TEC and DMC values will increase steadily for all three scenarios. However, the DMC in HVS shows a sharp increasing trend because of the relatively higher coefficient of the remittance compared to that of the TEC model. Given the remittance inflow trend over the last four decades, the range of TEC can be expected to be 21.5–29.6 billion koe, and DMC can be expected to be 251.6–378.1 million ktons, which are the ranges for the ETS and HVS values. However, given policies to reduce biomass and fossil fuel consumption (which constitutes most energy and material consumption) by improving the efficiency of use and moving towards a renewable energy transition, the TEC and DMC values may approach the LVS scenario. In 2017, the biomass share of the total DMC in Nepal was 74%, compared with almost 97% in 1985 and 93% in 2000 (Fig. 5). Similarly, the biomass share of the total TEC in Nepal was 73% in 2017, compared with 96% in 1985 and 87% in 2000. An increase in the use of fossil fuels compensated for the decreasing share of biomass in the TEC (Fig. 6). Fossil fuels’ share in TEC was less than 4% in 1985 and about 12% in 2000. It is now almost a quarter of the TEC.

### Government policies’ framing of climate mitigation actions and the contextual factor

Semi-structured interviews and a review of chosen government policies found that climate mitigation actions are framed into government policies in Nepal. However, the extent and social context in which framing is done during policy formulation and the priority for implementation vary across policies. A relatively strong framing of climate mitigation actions into policies of energy and forest sectors implies that government policymakers are inclined to increase access to renewable energy technologies and strengthen the carbon sink potential of the forest that covers about 40% of Nepal’s land area (Ministry of Population and Environment MoPE 2016a). In the first NDC that Nepal submitted in 2016, eight out of fourteen targets related to climate mitigation in energy and forest sectors (Ministry of Population and Environment MoPE 2016a). Likewise, the second NDC of Nepal submitted to the UNFCCC in 2020 focuses extensively on climate mitigation in the energy, forest, and transport sectors. Surprisingly, the 2015 Agriculture Development Strategy of an agriculture sector that contributes more than half of the nation’s GHG emissions has very few subjective statements on climate mitigation. This situation can be related to the social context of policymaking that sheds light on government policymakers’ preferences (interests) and their institutions’ position in the hierarchy of the government structure. The respondents from government organisations mentioned that the energy, forest, and transport sectors have more potential to reduce GHG emissions, have more financially viable projects, and significantly more co-benefits of climate mitigation actions compared with the agriculture sector of Nepal. The GHG emissions from the agriculture sector in Nepal come from livestock, rice cultivation, and managed soil (Thakuri et al. 2020). In lieu of reducing GHG emissions from the aforementioned sources in the agriculture sector, improving access to clean and renewable energy technologies, enhancing the carbon sink by protecting the forest area, and replacing fossil fuels with electric vehicles all present better co-benefits and appear to be more financially viable according to the respondents from the government organisations and frequently feature as aims in the chosen twelve policies. While this research did not find any evidence to corroborate the claim that the agriculture sector of Nepal has less potential to reduce GHG emissions, we found that co-benefits and local benefits are presented as important features of government policies.

Another contextual factor identified by this research is that global environmental discourse influences the contents of policies, including policy instruments (information, market, and economic instruments). External events (e.g. international climate agreements) can change the policymaking context (Moat et al. 2013). External forces, such as global environmental discourse and the associated international development mechanism, can dictate the policymaking process, including policy formulation, by influencing the knowledge, values, and interests of policy actors (Lovri et al. 2018). The policy actors’ ideas and the discourse pertaining to any agenda are also key to influencing decision-making whilst formulating policies (Carstensen and Schmidt 2016). This seems to be the case in Nepal, where the global environmental discourse is shaping and contributing to the framing of the climate mitigation actions into sectoral policies. Aryal et al. (2021) identified a similar trend, where international development organisations were found to play a critical role in shaping environmental policies in Nepal. Further, Laudari et al. (2021) and Baniya Baniya et al. (2021a) found that ODA Nepal receives as foreign aid from international development agencies active within the climate change domain have influenced the content of climate-specific national documents such as the NDC. The majority of respondents highlighted the collaborative financial partnership with international development agencies as key to framing climate mitigation actions. While most respondents viewed ODA as a source of funding for enabling local climate actions (including adaptation) at a community level, they have been used mostly for policymaking. The respondents from international development agencies refer to the limited institutional and technical capacities as issues needing attention during the framing process. Hence ODA is utilised in policymaking (whilst framing climate mitigation actions). We identify this as a source of conflict between the government agencies and the external forces, particularly given that both parties see ODA as a collaborative partnership tool for development.

The external forces driving the framing of climate mitigation actions in Nepal can be regarded as good as long as they are nationally relevant, are financially and technically feasible and, more importantly, fulfil the purpose of including climate mitigation actions in government policies. The policies of energy, forest, transport, and agriculture sectors have emphasised collaboration with an international development organisation, and the private sector and non-government organisations, to deliver their climate mitigation objectives. The diversity of different organisations involved, the discourses and collaborative practice amongst policy actors and their institutions, can all improve the credibility of government policies (Buijs et al. 2014; Baniya et al. 2021b). We found that this diversity-focused policy milieu is the social process that has created a preliminary climate mitigation-related knowledge structure, which is a part of the cognitive influence (Biermann and Siebenhüner (2009) of the global environmental discourse. Consequently, the fledgling knowledge structure is reflected in government policies, which we understand as framing climate mitigation actions, and this is a part of the normative influence.

### Policy actors’ ideas and institutions in framing and delivery of climate mitigation actions

Most respondents reported that strategic interactions between policy actors’ institutions are critical in the deliberative environment for framing cross-cutting issues like climate mitigation into government policies. In discursive institutionalism, where policy actors’ ideas and their institutions’ viewpoints on policy issues are exchanged, these interactions enrich the discourse and strengthen the analytical lens through which the influence of external and internal factors on policy change is better understood by policy actors (Buijs et al. 2014). Consequently, the government policies materialise discourse, policy actors’ ideas, and the institutional viewpoint. The shared policy beliefs and the historically constructed ‘cognitive beliefs’ of individual and collective policy actors can shape the decision-making (Goldstein and Keohane 1993). Similarly, their shared policy beliefs and ideational constructs can shape policy actors’ decision-making while the policy goes through changes (Béland 2016). In the case of Nepal, we found that policy actors discuss the framing of climate mitigation actions, policy problems, policy instruments, financial mechanisms, and institutional capacity as part of in-country deliberation of global environmental discourses.

Our research findings suggest that the policy actors’ technical and interpretative capacity is strengthened via engaging with international development agencies. Thus global environmental discourse advances within the national policy landscape. However, we also found that the discourse is more relevant to the central government policy actors than to the local government and private sector policy actors, who appeared to be more engaged in the implementation of climate mitigation actions on the ground. Therefore, in terms of materialising policy actors’ ideas in policymaking, central government policymakers and their institutions are more critical. In environmental policymaking, some policy actors may get limited space to raise their concerns even if they are included as key participants in deliberation for potential policy avenues (Aryal et al. 2020). This seems to be the case for Nepal, where the directionality of interactions in multi-level decision-making stems from central government organisations and extends vertically to the local government organisations. While this can be regarded as a participatory exclusion or deinstitutionalisation of policy actors at the local level (Agarwal 2001), the respondents from central government organisations referred to the way policy actors at different government levels deliver different responsibilities. In discursive institutionalism, where limited policy actors and their institutions are at the forefront, the favourable result of the deliberation may depend on the cognitive capacity of the participating policy actors. However, de-emphasising the role of the policy actors’ institutions in policymaking is also a part of normative policymaking (Carstensen 2011; Wood 2015). Therefore, undermining the role of local government organisations whilst operating at the intersection of global environmental discourse and national policies by the central government policymakers can be a part of normative policymaking. However, despite having a minor role, local government organisations are found to participate extensively in implementing climate mitigation actions, which this research identifies as a factor that may lead to delivering climate mitigation actions.

While the ideas, interests, and institutions of policy actors that are key to driving changes in policies (Walt 1994) appeared to be important in the framing of climate mitigation in Nepal, the collaborative practice between policy actors shed light on creating an effective policy network^4^. This research found that collaborative practice occurs within the policy network realm, which is considered to be variable in the sense that it is likely to change in response to one institution’s pressures over others (Shearer et al. 2016). Whilst framing climate mitigation actions via government policies in Nepal, the respondents did not report a history of fluidity in the policy network in the context of climate-specific and sectoral policies. However, this research found that the interaction between central and local government organisations and the way they exercise their power differently have created conflicting responsibilities regarding the framing and delivery of climate mitigation actions in Nepal. This is usually a case when the policy network consists of either informal institutions or weak institutions (Helmke and Levitsky 2004). The local level government organisation in Nepal appeared to be weak, particularly in policy formulation. However, since the policymaking process leverages a relational approach to involving multifarious institutions and actors while creating a policy network, some actors can be reactive while the lead institution can be a facilitator (De Marchi et al. 2016; Aryal et al. 2021). This research found that the environment sector (e.g., environment ministry) is the lead institution that creates a network of limited policy actors, reinforces relations among policy actors, and delegates’ responsibilities to the local level government organisations to deliver climate mitigation actions. While the facilitation by the lead institution is supposed to enhance the collaborative practice between policy actors, availability and equitable distribution of resources (e.g. financial resources) may determine if the collaborative practice can materialise the framing of climate mitigation actions in Nepal.

### Understanding the delivery prospects of the climate mitigation actions in Nepal

Framing ambitious climate mitigation actions into government policies does not necessarily mean that they are achievable (Pauw et al. 2019). The projection of TEC, DMC, and GHG emissions indicate that delivering climate mitigation actions at an economy-wide scale is challenging, particularly in the absence of policies focusing on the efficiency of biomass and fossil fuel use and their absolute reductions. This finding shed light on the importance of the following: 1) creating policy alignment^5^ between climate-specific policies (Climate Change Policy and NDCs) and sectoral policies in Nepal (Baniya et al. 2021a); 2) leveraging the NDC preparatory process during which climate mitigation actions are determined and deliberated for framing; and 3) strengthening the sectoral foci of climate mitigation actions—criticised by Regmi and Bhandari (2013) and Ojha et al. (2016)—but presenting better prospects of delivery in the case of Nepal.

This research found that the policy alignment is relatively strong between climate-specific policies and the energy and forest sector policies. This finding corresponds to the study of Shrestha and Dhakal (2019) who state that climate mitigation efforts are directed mainly towards energy policies and REDD+ in Nepal. It means the prospect for delivering climate mitigation actions are either analysed only for priority sectors (e.g., energy, forest, and transport) or done for all but insufficiently considered. Insufficient consideration of climate mitigation targets, particularly in the NDC preparatory process during which sectoral commitments are also deliberated and determined, may lead to implementation problems (Röser et al. 2020). Most respondents identified the NDC preparatory process to be critical in framing climate mitigation actions in government policies, which determines their delivery prospect. Nevertheless, we find that policy actors in Nepal were unable to leverage the NDC preparatory process which occurs once every five years, prior to the NDC submission to the UNFCCC. Both NDCs submitted to UNFCCC in 2016 and 2020 have excluded energy efficiency measures and targets, and quantitative mitigation targets for the agriculture sector. Therefore, while the policy alignment is strong for the policy of the energy sector, it is also insufficient. On the other hand, policy alignment is weak for the agriculture sector. This may have resulted from government policies’ emphasis on co-benefits. For example, sustainable agriculture practices, sustainable transportation, and reducing indoor air pollution from biomass as a cooking fuel are presented as subjective policy statements without any quantitative targets aimed at reducing GHG emissions.

The notion of NDCs’ sectoral foci and the related debate stems from studies that criticise the portrayal of climate change as a sectoral agenda in Nepal, which limits the potential for politicisation of climate change issues (Ojha et al. 2016; Laudari et al. 2021). While the criticism of technocratic hegemony is justified given the coordination between line ministries (sectors) in NDC’s preparatory process, it is not a huge concern for most countries (Röser et al. 2020). This research identifies three key factors— technical, structural, and material—that are not socio-political yet may determine the successful delivery of climate mitigation actions. Although the respondents mentioned using a technocratic approach to determining climate mitigation targets across sector policies, this research did not identify a strong techno-bureaucratic influence whilst framing the climate mitigation actions as policy goals. The interviews with respondents revealed that the relevant bureaucrats and non-government policy actors had both educational and professional backgrounds in social science and economics, meaning techno-bureaucratic influence is minimal. Nonetheless, the majority of respondents concur that climate issues (both mitigation and adaptation) have received limited political attention and the local climate governance – especially regarding climate mitigation – is still a work in progress as the country’s governance and administration transition into the federal structure. Further, the predicted values of TEC, DMC, and GHG emissions may hold true because the energy sector and agriculture sector policies that contribute more than 85% of the nation’s GHG emissions collectively lack sufficient climate mitigation actions. This is a technical issue that the policy alignment and the subsequent NDC preparatory process have ignored, despite the respondents from government organisations stating that a technocratic approach complements the discursive policymaking, and that the energy sector is the prioritised sector via which climate mitigation actions are planned to be delivered.

The structural factor pertains to the structural feature of an economy and the associated GHG emissions. While the agriculture, forestry, and fishery sector contribution to GDP in Nepal has been in decline for the last 15 years, GHG emissions are still projected to increase for all scenarios, and the agriculture sector will still be significant because of the low negative standardised coefficient of the ‘agrishare’ (Table 3). The Third National Communication (Ministry of Forest and Environment MoFE 2021) submitted by the Government of Nepal to UNFCCC projects a marginal increase in GHG emissions from the agriculture sector (including forestry and land use) despite its reduced share in generating the nation’s GDP. Nonetheless, the absolute GDP from the agriculture, forestry, and fishery sector has increased by almost four-fold in the last two decades (World Bank 2021). Therefore, while it may appear that the reduced share of agriculture, forestry, and fishery sector in Nepal’s GDP have the least bearing on its sectoral GHG emissions, the absolute GDP – four-fold increase – is significant enough to consider this sector as needing appropriate climate mitigation actions. Ignoring this structural feature of Nepal’s GHG emissions (Baniya et al. 2021c) will present challenges in terms of delivering climate mitigation actions collectively across energy and agriculture sectors. The respondents and the Second National Communication (Ministry of Science Technology Environment MOSTE 2014) hinted that Nepal’s agriculture sector (excluding forestry and land use) has limited climate mitigation opportunities. However, we suggest that policymakers take into account the economy’s structural changes and the related implications on GHG emissions from both the energy and agriculture sector (excluding forestry and land use) whilst framing climate mitigation actions across government policies.

Finally, the material factor, which refers to human and financial resources, is another critical aspect of linking climate mitigation actions to the sector policy goals. Institutional constraints, such as limited human and financial resources, have often weakened the prospect of achieving policy alignment, particularly in developing countries (Atteridge et al. 2019). Many low-income and developing countries are already struggling to access technical and financial support to implement their NDCs (Pauw et al. 2019). This is corroborated from the semi-structured interviews in which respondents talked about the competition between government institutions to access limited financial resources, and there are limited active on-ground projects that are delivering climate mitigation actions in Nepal (e.g., small-scale renewable energy technologies). International development agencies are increasingly pledging climate change-related funds, but they have tended to turn away without much contribution (Mahat et al. 2019). While this has encouraged policymakers to diversify climate mitigation-related finance options, most respondents stated that the delivery prospect is beset by the lack of sufficient climate mitigation-related finance. The respondents from the government organisations mentioned increasing allocation of climate mitigation-related finance from government sources but did not mention leveraging the immense potential to mobilise the remittances, given its one-fourth share in the GDP in 2020 (World Bank 2021). Das et al. (2021) have identified a long-run causality between remittance and renewable energy consumption, meaning that the remittance can be used to stimulate household-level renewable energy technologies that can replace biomass and fossil fuels to some extent.

## Conclusion

This research generated three key insights into the linkage between the framing of climate mitigation actions into government policies and the prospect for their delivery. First, the structural change in the economy, with the reduced share of GDP from the agriculture, forestry, and fishery sector driving shrinkage of GHG emissions from this historically significant sector. However, the rapid increase in energy consumption per capita, driven primarily by the remittance and the GDP per capita, is set to be an issue in the longer term. Owing to the small share of hydroelectricity in Nepal’s energy mix (Fig. 6), this research suggests that energy efficiency measures—that remain ignored by the recently submitted NDC—and renewable energy technologies need to be deployed to achieve absolute reductions in the use of biomass and fossil fuels, and the related GHG emissions. Biomass that contributes to the majority of domestic material consumption (Fig. 5) and the total energy consumption (Fig. 6) is a low-intensity energy resource, meaning poor efficiency in terms of both use and conversion. Thus, biomass substitution together with fossil fuels by small-scale modern renewable energy technologies like solar PV, improved cook stoves, biogas, and micro-hydro will reduce material consumption and the related GHG emissions whilst improving the energy efficiency that Nepal’s NDCs have excluded. The rural low-income households that often have poor access to high quality energy (e.g. electricity and biogas) for lighting and cooking will benefit from small-scale modern renewable energy technologies as they currently depend extensively on low quality energy resource biomass. The small-scale renewable energy transition is being enabled in rural areas of Nepal (Fig. 6) mostly because of government subsidies for rural energy, relatively low cost, and remittances (Das et al. 2021; Baniya and Aryal 2022). In fact, to promote energy equality, the Government of Nepal have targeted to reduce biomass consumption by half in 2030 relative to 2015 (National Planning Commission NPC 2020) whilst envisioning small-scale modern renewable energy sources in low-income rural areas.

Second, this research found that the policy alignment, the NDC preparatory process, and the climate mitigation actions’ sectoral foci have been inclined towards Nepal’s energy, forest, and recently transport sectors. However, insufficient climate mitigation actions in the energy sector and the structural issue that ignored climate mitigation actions in the agriculture sector highlight a need to overcome policy silos that result when the act of framing does not sufficiently consider the delivery prospect. In circumstances like this, when there is a need to reinforce synergies between goals of sector policies, better policy alignment is suggested, not only to overcome silo thinking but also to integrate issues from one policy domain to another. The specific policy suggestion is to explore limited climate mitigation opportunities within the agriculture sector that respondents mentioned about, and initiate sufficient actions (e.g. energy efficiency that current NDCs lack) within the energy sector. As the energy, forest and transport sectors are identified as potential sectors for climate mitigation actions, these sectors should have sufficient mitigation actions to cover up for the agriculture sector, which is deemed as having relatively low potential to reduce GHG emissions.

Third, while the policymakers mentioned diversifying the climate mitigation-related finance, the potential role of remittance in stimulating household-level small-scale modern renewable energy technologies seems to be ignored, given the contribution of remittance to the economy. Our findings suggest that remittance will be a key determinant of future TEC, DMC, and GHG emissions. Therefore, considering financial constraints that challenge the possibility of delivering the climate mitigation actions into policies, the role of remittance can be investigated in detail. Based on the three insights, we conclude that the framing of climate mitigation actions and the possibility for their delivery is fragmented. Nonetheless, the consideration of climate mitigation actions in policy formulation can be extended up to policy implementation whilst dealing with the factors that caused fragmentation and policy silos.

## Supplementary Information

Supplementary material

## Data Availability

The datasets generated during and/or analysed during the current study are available from the corresponding author on reasonable request.

## Footnotes

1 Non-policy actions are intended for implementing the climate mitigation objectives of the government policies and are not directly linked to policy formulation.

2 Resource efficiency means an efficient use of the resources while protecting the environment, for example, by creating more value from fewer resources as an input while also considering the ecological limits and regenerative capacity of natural resources. All forms of energy resources are typically referred to as resources in this paper.

3 Domestic material consumption measures the total amount of materials directly used by an economy and is calculated as the annual quantity of raw materials extracted from domestic territories, plus all physical imports minus physical exports (Eurostat 2021). In this paper, DMC includes biomass, fossil fuels, metal ores, and construction materials.

4 Policy network refers to the policy actors’ group that forms a network to interact during policymaking.

5 In this research, policy alignment is understood as the process of aligning economic sector policy goals with the climate mitigation objectives
