# Supplementary material for: Can the Framing of Climate Mitigation Actions into Government Policies Lead to Delivering Them? – Insights from Nepal’s Experience
Source: Environ Manage. 2022 Apr 23;70(2):179–200. doi: 10.1007/s00267-022-01643-6 (PMC9034080; doi:10.1007/s00267-022-01643-6)
Supplement: Supplementary file 1 — Supplementary material [file 267_2022_1643_MOESM1_ESM.pdf]

Table 1. Qualitative methods, their use, elements covered, and data sources.

Qualitative methods	Description and purpose in the context of this research	Elements studied by this research	Data sources
**Critical discourse analysis (CDA)**	- ***Description*** – It sees the use of language (e.g., written texts such as policy documents) as social practice and dialectical relationship between institutions.<br><br>- ***Purpose*** – To interpret findings in a broader social context and seek to explain the social process and factors around the framing of climate mitigation actions.	1) discursive differences.<br>2) preferential values of policy actors.<br>3) differing ideologies and ideas in general.<br><br>**(FACTORS)**	Government policy documents (12)
**Post-structural discourse analysis (PDA)**	- ***Description*** – It focuses on the interaction between policy actors and their institutions and related discourse within the public policy realm.<br><br>- ***Purpose*** – To help understand the power dimension of the discourse and the intersubjective domain of ideational exchange between policy actors and their institutions.	1) interest based discourse<br>2) ideational power.<br>3) directionality of institutions.<br><br>**(INTERACTION)**	Government policy documents (12) and semi-structured interviews (12).
**Thematic analysis (PA)**	- ***Description*** – It segregated textual data corpus into themes for deductive reasoning.<br><br>- ***Purpose*** – The purpose of thematic analysis is to decode both explicit and implicit meanings of the textual data corpus and interviews narration into three main themes that this research uses for deductive reasoning.	1) institutional, financial, and technical capacities.<br>2) determinants for delivery of climate mitigation actions.<br>3) Stakeholders buy-in.<br><br>**(ENABLERS)**	Semi-structured interviews (12).
